# Similia Not Idem

**Published:** 1882-01

**Authors:** 


### SIMILIA NOT IDEM.

### HAHNEMANN'S OPINION.

"The Homœopathic doctrine has never taught to cure a desease by the very same agent which had produced it; this has been repeated over and over again, although to all appearances in vain. Homœopathy professes to cure diseases by means of drugs which produce exactly similar, but not identical, symptoms." (Foot-note to Hahnemann's reply to his critics).

